# T1 mapping: characterisation of myocardial interstitial space

**DOI:** 10.1007/s13244-014-0366-9

**Published:** 2014-11-26

**Authors:** Rosario J. Perea, Jose T. Ortiz-Perez, Manel Sole, M. Teresa Cibeira, Teresa M. de Caralt, Susanna Prat-Gonzalez, Xavier Bosch, Antonio Berruezo, Marcelo Sanchez, Joan Blade

**Affiliations:** 1 Radiology Department. Hospital Clinic, University of Barcelona, Barcelona, Spain; 2 Cardiology Department. Hospital Clinic, University of Barcelona, Barcelona, Spain; 3 Pathology Department. Hospital Clinic, University of Barcelona, Barcelona, Spain; 4 Haematology Department. Hospital Clinic, University of Barcelona, Barcelona, Spain

**Keywords:** Magnetic resonance imaging, T1 mapping, Extracellular volume fraction, Diffuse myocardial fibrosis, Interstitial space

## Abstract

**Abstract:**

Myocardial fibrosis is always present in end-stage heart failure and is a major independent predictor of adverse cardiac outcome. Cardiac magnetic resonance (CMR) is an imaging method that permits a non-invasive assessment of the heart and has been established as the “gold standard” for the evaluation of cardiac anatomy and function, as well as for quantifying focal myocardial fibrosis in both ischaemic and non-ischaemic heart disease. However, cardiac pathologies characterised by diffuse myocardial fibrosis cannot be evaluated by late gadolinium enhancement (LGE) imaging, as there are no reference regions of normal myocardium. Recent improvements in CMR imaging techniques have enabled parametric mapping of relaxation properties (T1, T2 and T2*) clinically feasible within a single breath-hold. T1 mapping techniques performed both with and without contrast enable the quantification of diffuse myocardial fibrosis and myocardial infiltration. This article reviews current imaging techniques, emerging applications and the future potential and limitations of CMR for T1 mapping.

**Teaching points:**

• Myocardial fibrosis is a common endpoint in a variety of cardiac diseases.

• Myocardial fibrosis results in myocardial stiffness, heart failure, arrhythmia and sudden death.

• T1-mapping CMR techniques enable the quantification of diffuse myocardial fibrosis.

• Native T1 reflects myocardial disease involving the myocyte and interstitium.

• The use of gadolinium allows measurement of the extracellular volume fraction, reflecting interstitial space.

## Introduction

The normal myocardium is composed of cardiac cells, blood vessels and nerves embedded within a complex three-dimensional space, the interstitium or extracellular space. The interstitium is a complex and dynamic environment, vital for normal cardiac structure and function. In the normal human heart, the extracellular matrix (ECM) is predominantly made up of collagen scaffolding [1, 2] and contains a ground substance of proteoglycans and glycosaminoglycans, as well as fibroblasts and immune cells [3]. One of the distinctive factors of its pathology is interstitial space expansion, normally through the development of fibrosis. Myocardial fibrosis is associated with worsening ventricular function, abnormal cardiac remodelling and increased ventricular stiffness [4]. Moreover, fibrosis plays an important role in the development of arrhythmia and sudden death [5], having been shown that it is an independent predictor of major adverse cardiac events (heart failure, arrhythmia and death) [6].

Currently, the only method to quantify diffuse fibrosis is invasive biopsy, which carries significant morbidity, is prone to sampling error and fibrotic involvement of the whole left ventricle cannot be determined [7]. Blood biomarkers for fibrosis assessment are also known to have complex confounding factors. Late gadolinium enhancement (LGE) imaging with cardiac magnetic resonance (CMR) has been the “gold standard” for detecting focal myocardial fibrosis in clinical practice. While LGE is clinically useful [8], reliance on relative signal intensity changes and nulling of “normal appearing” myocardium make it difficult to identify subtle abnormalities such as diffuse interstitial fibrosis [9]. A unique feature of CMR is its ability to use proton relaxation times, such as T1 to characterise myocardial tissue [9]. These relaxation times can be quantified using recently created mapping sequences [10, 11].

This article describes the emerging techniques of myocardial T1 mapping and extracellular volume quantification, evaluates its capacity to characterise myocardial tissue and demonstrates its clinical relevance.

## Etiophysiopathology of myocardial fibrosis

The healthy myocardium contains an ECM that is a major determinant of its structural integrity and mechanical functions [3]. Normally, the ECM and fibrillar collagen network form only 6 % and 2-4 %, respectively, of the structural space within the heart [12]. However, the interstitium is actively maintained by the relationships between itself, myocytes, the neurohormonal system, mechanical forces and cardiac fibroblasts [13]. Within these coexisting matrices, a constant flux of tissue and collagen turnover takes place, coordinated by regulatory cytokines, growth factors, enzymes, hormones and direct cell-to-cell communication [14].

Diffuse myocardial fibrosis is a covert process that occurs as a part of normal ageing [8] but is accelerated in disease [15, 16, 17, 18]. Although fibrotic remodelling is not completely understood, after a specific cardiovascular stress (e.g. an ischaemic or mechanical injury), a cascade of chemokines, cytokines, neurohormonal factors and matrix metalloproteinases lead to local cell activation and collagen synthesis [19]. Myocardial fibrosis, defined as a significant increase in the collagen volume fraction (CVF) of myocardial tissue, is a common endpoint in a variety of cardiac diseases [20]. The distribution of myocardial fibrosis, however, varies according to the underlying pathology [20]. The progressive accumulation of collagen develops a range of ventricular dysfunctional processes that generally affect diastolic and subsequently systolic function [4]. Usually, myocardial fibrosis is classified as interstitial or replacement fibrosis.

**Fig. 1:**
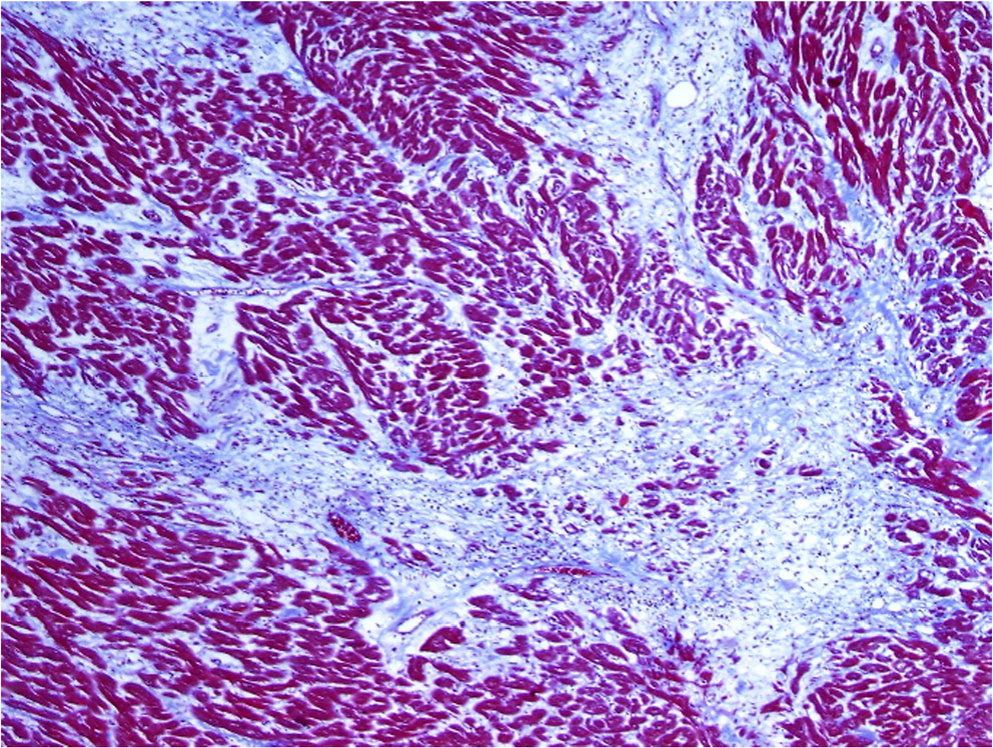
Scarring or replacement fibrosis after myocardial infarction: a stellate fibrous scar (*blue areas*) replaces myocardial parenchyma (Masson trichrome staining ×40)

Replacement or scarring fibrosis corresponds to the replacement of myocytes after cell damage or necrosis by plexiform fibrosis [21]. It may have localised (Fig. 1) (ischaemic cardiomyopathy, myocarditis, hypertrophic cardiomyopathy and sarcoidosis) or diffuse distribution (chronic renal insufficiency, toxic cardiomyopathies and inflammatory diseases) depending on the underlying aetiology. The most common cause of replacement fibrosis is scarring from myocardial infarction. LGE is a validated way to identify focal replacement fibrosis [8].

**Fig. 2:**
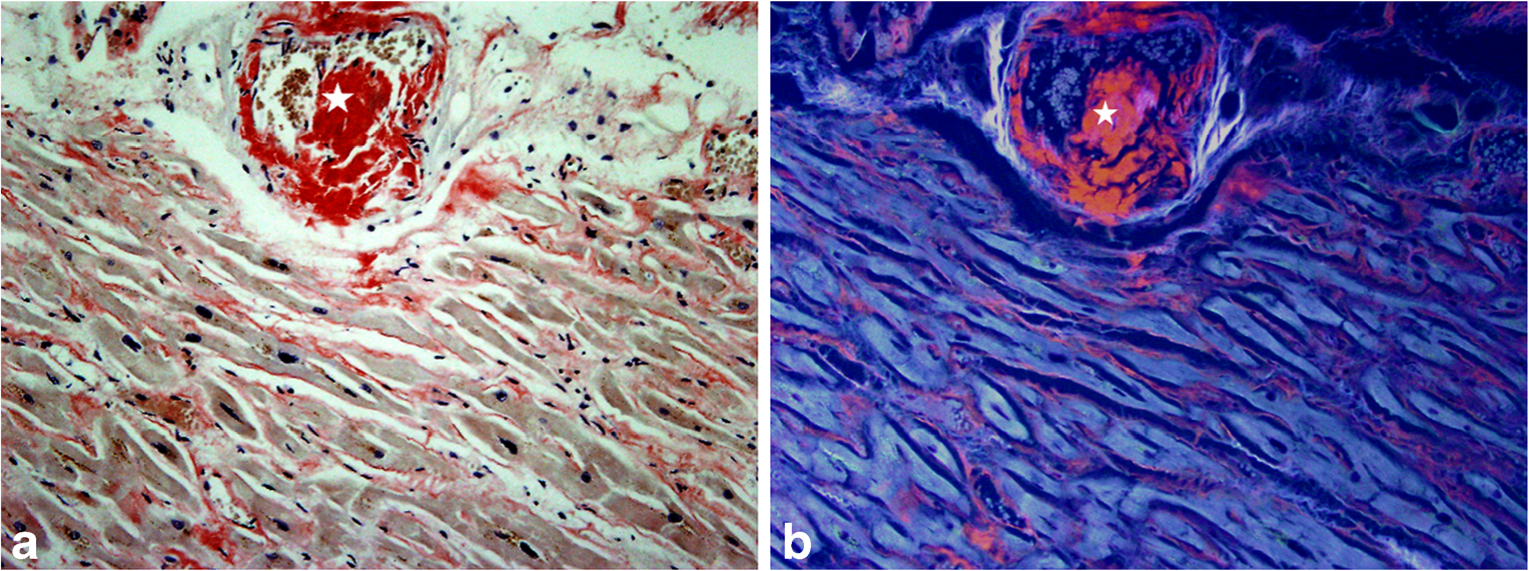
Cardiac AL amyloidosis with interstitial (perimyocytic) and vascular involvement (*star*). (Congo red stain with **a** normal light and **b** ultraviolet light microscopy ×100). *Red areas* represent amyloid deposition

Interstitial fibrosis has a diffuse distribution within the interstitium and its subtypes include reactive and infiltrative fibrosis. Reactive fibrosis has a progressive onset and follows the increase in collagen synthesis by myofibroblasts under the influence of different stimuli. It has mostly been described in hypertension [15] and diabetes [16], but it is also present in the ageing heart [22], in idiopathic dilated cardiomyopathy [23], and in left ventricular pressure-overload and volume-overload states induced by chronic aortic valve regurgitation and stenosis [24]. It has also been reported in the remote non-infarcted myocardium after infarction [18]. Infiltrative fibrosis is more unusual and is induced by the progressive deposit of insoluble proteins (amyloidosis) [25] (Fig. 2) or glycosphingolipids (Anderson-Fabry disease) [26] in the cardiac tissue. Their pathophysiology follows similar patterns as reactive fibrosis. Interstitial fibrosis precedes irreversible replacement fibrosis [27]. Reactive and infiltrative fibrosis may be reversible under specific therapy [28]. Therefore, the early detection of cardiac involvement is of critical importance to therapeutic management.

## Detection of myocardial fibrosis

**Fig. 3:**
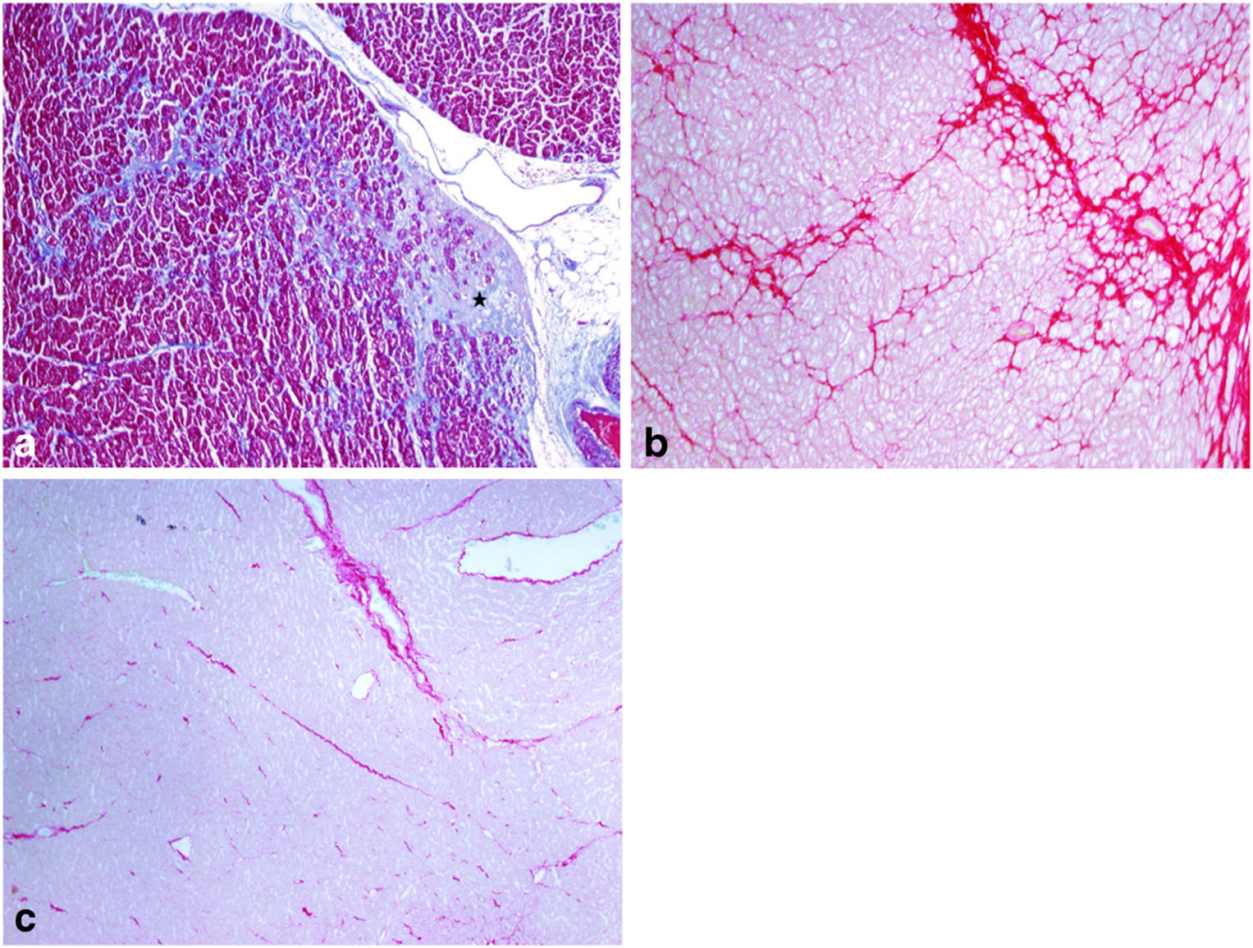
Dilated cardiomyopathy of toxic origin with myocardial focal replacement containing fibrous tissue (*star*) and interstitial widening for fibrosis (Masson trichrome staining). *Blue areas* depict fibrosis, *red areas* are cardiomyocytes. (**b**) Picrosirius red stain enhances the perimyocytic pattern of fibrosis (*red areas* represent fibrosis). (**c**) Picrosirius red stain of normal myocardium for comparison. (Original magnification ×40)

Until now, the only approach to assess myocardial fibrosis has been endomyocardial biopsy. This methodology allows qualitative macroscopic assessment with Masson Trichrome staining [29] and quantitative morphometry (quantification of CVF) with picrosirius red [30] (Fig. 3). However, this technique is invasive and prone to sampling errors. CMR T1 mapping of the myocardium has the potential to quantify myocardial fibrosis in a non-invasive way. Preliminary studies suggest that these techniques are reproducible and may be more reliable than the current biopsy gold standard, because the biopsy sample represents less than a thousandth of the total myocardial volume. Furthermore, these techniques can potentially quantify the fibrosis of the whole heart, which truly reflects the global myocardial fibrosis burden. These new biological parameters have the ability to detect early disease, guide therapy and predict outcomes [31].

## Myocardial T1 mapping

Quantitative myocardial T1 mapping is a CMR technique that provides in vivo tissue characterisation [10]

In CMR images, the pixel signal intensity is based on the relaxation of hydrogen nuclei protons in a static magnetic field. T1 relaxation time depends on the molecular environment of the water molecules in the tissue and therefore characterises each tissue very specifically. T1 relaxation time varies from one type of tissue to another, but also within the same tissue depending on its physiopathological status (inflammation, oedema, fat, fibrosis, etc.).

**Fig. 4:**
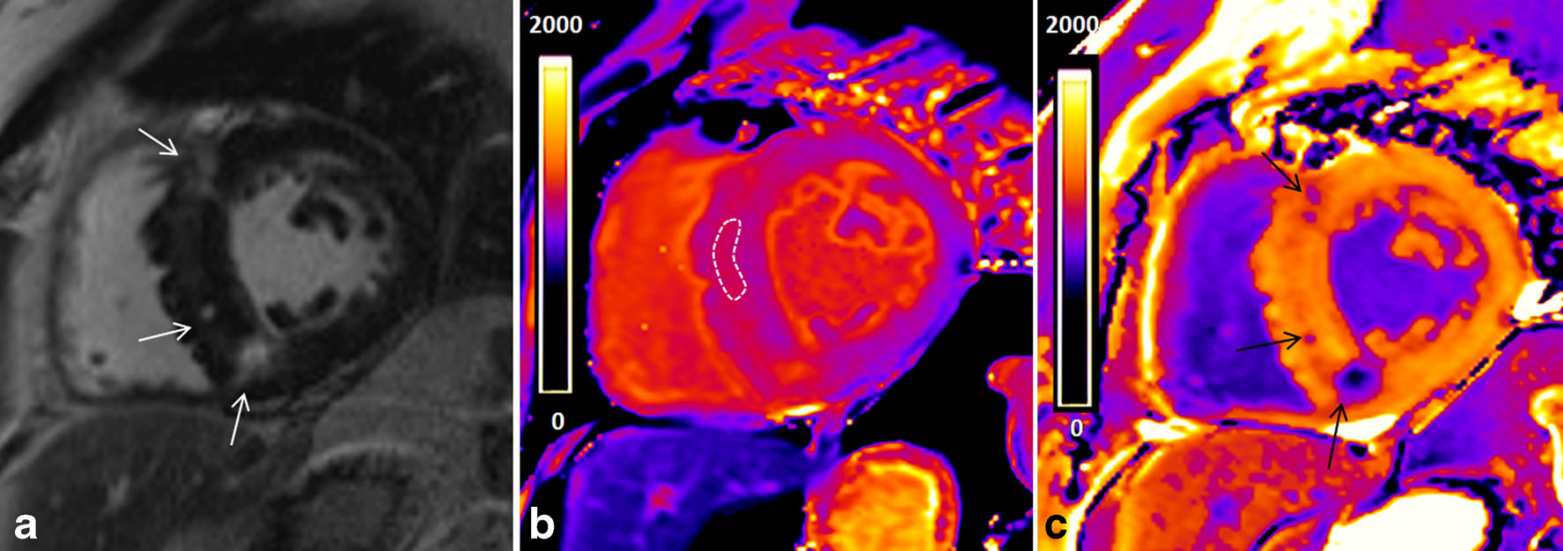
T1 mapping in hypertrophic cardiomyopathy. **a** LGE imaging demonstrating patchy mid-wall enhancement in the septum (*white arrows*). **b** MOLLI T1 map at 3 T (Magneton Trio-Tim; Siemens, Erlangen, Germany) demonstrating increased T1 value (1,161 ms) in an area without LGE (ROI) because of diffuse fibrosis. The T1 value in the area with LGE (focal fibrosis) is 1,281 ms. **c** Post-contrast T1 map illustrating excellent agreement between LGE (*black arrows*). Post-contrast T1 values are shortened in the area with late enhancement (301 ms) as well as in the rest of the septum (465 ms)

Gadolinium-based contrast agents (GBCAs) shorten T1. These low-molecular-weight extracellular agents are small enough to pass across the vascular wall into the extracellular space, yet are large enough that they do not penetrate cells with intact membranes. They accumulate passively in the gaps between cells and the increased volume of distribution of interstitial expansion in “scar” tissue [32]. This forms the basis of the LGE for detection of focal fibrosis and recent developments have built upon this, further allowing scrutiny of diffuse interstitial expansion (Fig. 4).

**Fig. 5:**
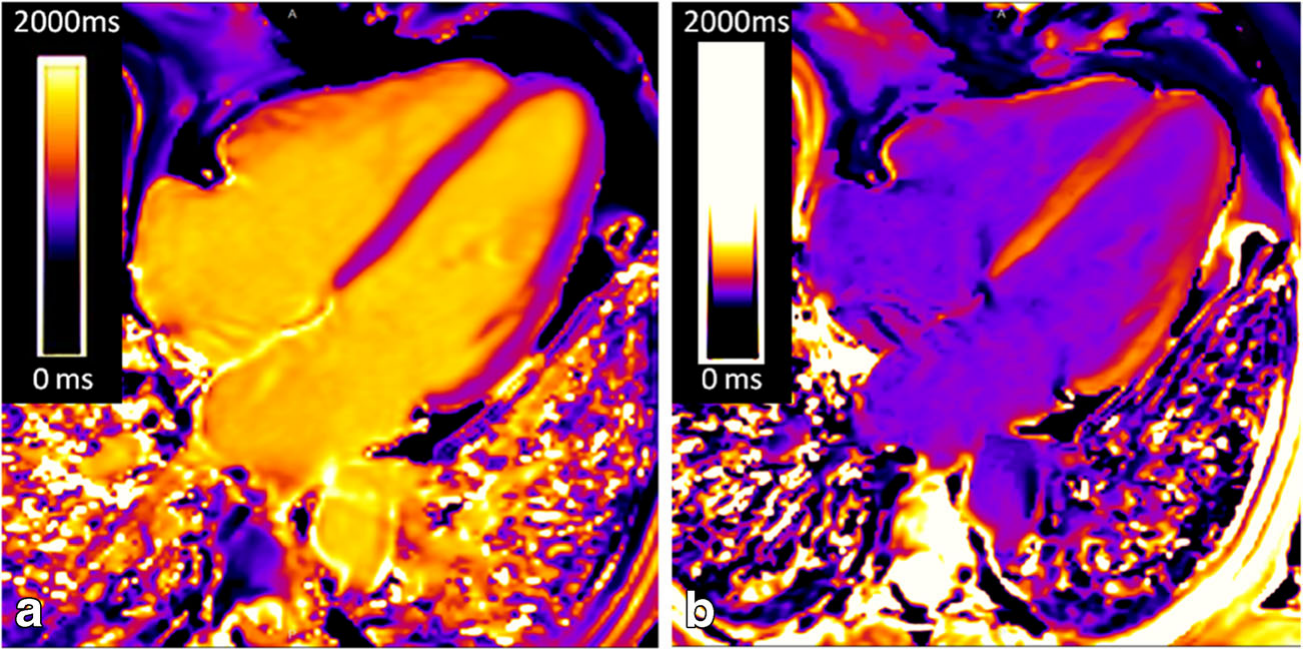
Quantification of native T1 values of a healthy volunteer in a four-chamber view obtained at 3 T. The resulting pixel by pixel colour native T1 map is displayed using a customised table where normal myocardium is *purple* and increasing T1 ranges from *orange* to *yellow*. Normal values of native T1 at 3 T (Magneton Trio-Tim; Siemens) with MOLLI sequence are 1,031 ± 24 ms. Post-contrast T1 values at 15 min are much shorter (557 ms, in the range of *orange* in the colour scale), due to the relaxing effect of the residual gadolinium in the myocardial interstitium

A T1 map of the myocardium is a parametric reconstructed image, where each pixel’s intensity directly corresponds to the T1 relaxation time of the corresponding myocardial voxel. Therefore, it allows signal quantification (in milliseconds) on a standardised scale of each myocardial voxel with high spatial resolution [10]. Compared with LGE images, T1 mapping CMR techniques eliminate the influences of windowing and variations in signal enhancement by directly measuring the underlying T1 relaxation times. Pre-contrast or native T1 times in normal myocardium are longer than post-contrast T1, due to the small amount of residual gadolinium in the myocardial interstitium (Fig. 5). Native and post-contrast T1 mapping can be performed to measure the extracellular volume fraction (ECV) [32], which has important prognostic value [31] and shows promise for the detection of diffuse myocardial fibrosis [33, 34]. These techniques will solve the problem of detecting the processes that diffusely affect the myocardium.

## T1 mapping methodology

### T1 mapping sequences

T1 maps originate from a series of co-registered images acquired at different times of T1 recovery, typically following a magnetisation preparation by inversion or saturation [35]. Raw images used for T1 mapping have to be acquired at identical times in the cardiac cycle. Different CMR acquisition sequences have been used to obtain myocardial T1 maps, including the modified Look-Locker inversion recovery (MOLLI) [10], shortened MOLLI (ShMOLLI) [36], saturation recovery single-shot acquisition (SASHA) [37] and saturation pulse prepared heart rate independent inversion recovery (SAPPHIRE) [38].

T1 measurements can be altered by several factors, such as the acquisition scheme, magnetisation transfer, flow, T2 effect and motion [35, 36, 37, 39, 40]. This is an essential point to consider before performing myocardial T1 maps, because it directly influences the accuracy and reproducibility of the final T1 measurements. This will also be considered when comparing results between different studies. Different T1 mapping strategies will have varying sensitivities to motion artefacts, heart rate, and intrinsic T1 values ranges [41]; Table 1 shows normal values of myocardial T1 mapping at different studies.

**Table 1:** Reference values of native myocardial T1 mapping at different studies

	Field strength	Scanner	Reception coil	T1 mapping sequence	T1 values (ms)
Messroghli et al. [42] 2006	1.5 T	Gyroscan Intera CV, Philips	–	MOLLI	980 ± 53
Piechnik et al. [36] 2010	1.5 T	Avanto, Siemens	32-channel	ShMOLLI	966 ± 48
	1.5 T	Avanto, Siemens	32-channel	MOLLI	976 ± 46
	3 T	Trio, Siemens	16-channel	ShMOLLI	1,166 ± 60
	3 T	Trio, Siemens	16-channel	MOLLI	1,169 ± 45
Lee et al. [58] 2011	3 T	Verio, Siemens	32-channel	MOLLI	1,315 ± 39
Rogers et al. [59] 2013	1.5 T	Philips, Best	32-channel	MOLLI	976 ± 37
	3 T	Philips, Best	32-channel	MOLLI	1,108 ± 67
Von Knobelsdorff et al. [49] 2013	3 T	Verio, Siemens	32-channel	MOLLI	1,158 (range, 1,005–1,295)
Kellman et al. [82] 2013	1.5 T	Avanto, Siemens	32-channel	MOLLI	1,012 ± 25
Piechnik et al. [83] 2013	1.5 T	Avanto, Siemens	16-channel	ShMOLLI	962 ± 25
	1.5 T	Avanto, Siemens	32-channel	ShMOLLI	962 ± 25
Puntmann et al. [74] 2013	3 T	Achieva TX, Philips	32-channel	MOLLI	1,070 ± 55
Fontana et al. [76] 2014	1.5 T	Avanto, Siemens	16-channel	ShMOLLI	967 ± 34
Liu et al. [84] 2014	3 T	Trio, Siemens	12-channel	MOLLI	1,232 ± 51

**Fig. 6:**
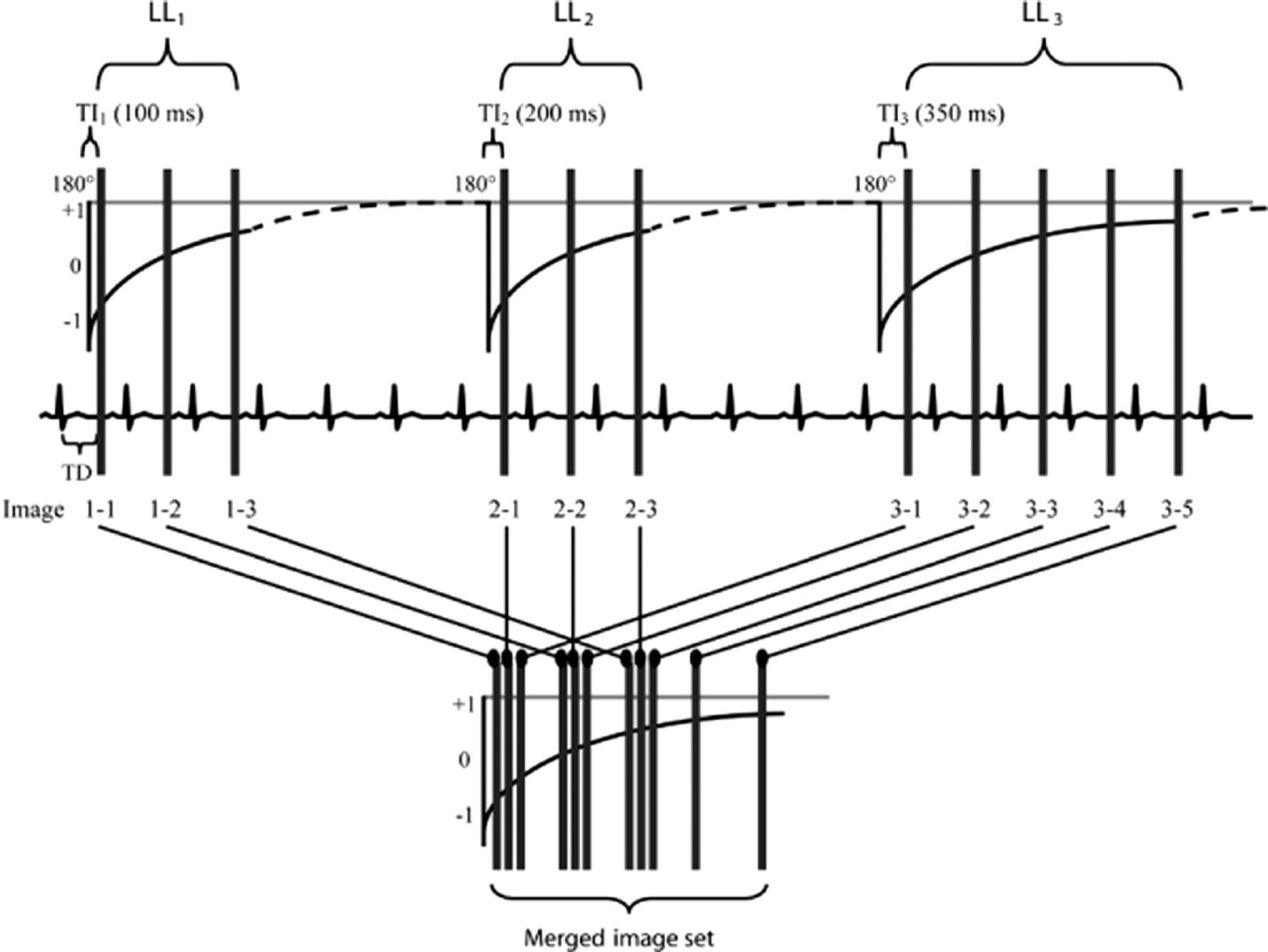
MOLLI pulse sequence scheme. *Vertical bars* represent image acquisition. *Dashed lines* represent undisturbed signal recovery. Three sets of LL experiments are performed successively with increasing TI within one breath-hold’s time. Images are acquired with a specific trigger delay (TD) to select end-diastole. For post-processing (calculation of T1 values), the images are regrouped into one data set according to their effective TI. (Reprinted, with permission, from Messroghli et al. [10])

**Fig. 7:**
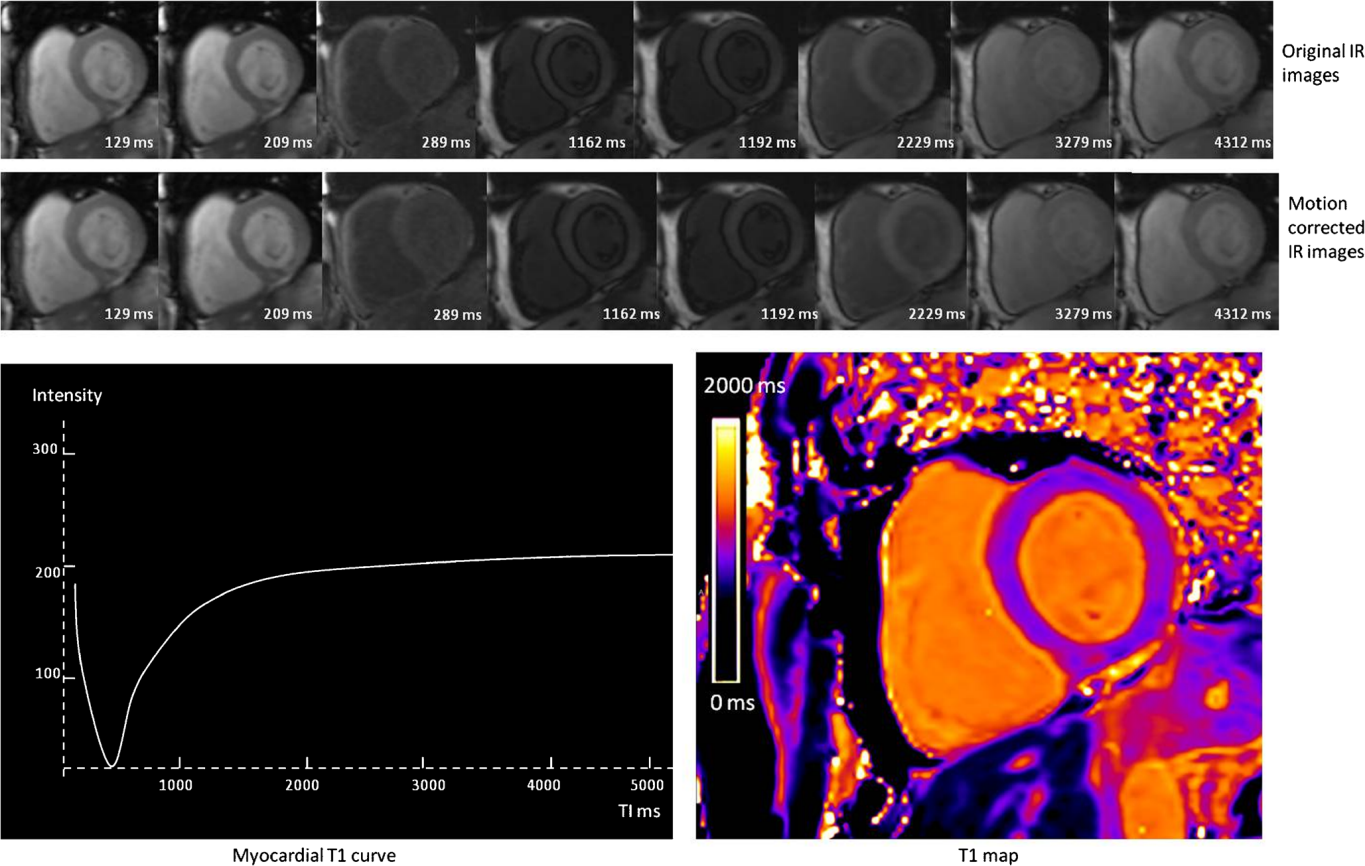
MOLLI T1 mapping in a healthy subject acquired at 3 T (Magneton Trio-Tim; Siemens). The *top row* shows the original images acquired at different TI times. The *bottom row* shows the motion corrected images using a non-rigid registration algorithm. By merging these source images into one data set, T1 values can be computed for every pixel with three-parameter curve fitting; a map of T1 in the imaging section can then be generated from these pixel values

The most assessed T1-mapping sequence has been described by Messroghli et al. [10, 41] and is the MOLLI sequence that provides high-resolution T1 maps of human myocardium. Although it is sensitive to extreme heart rate values and tends to slightly underestimate the true T1 value, the method allows a rapid and highly reproducible T1 map of heart with high levels of intra- and inter-observer agreement [42]. The MOLLI method [10, 41] overcame the limitations of motion and prolonged acquisition time of Look-Locker (LL) sequences [43], incorporating an efficient sampling of the T1 relaxation curve. The MOLLI sequence employs selective data acquisition at a given time of the cardiac cycle over successive heartbeats and merges data from multiple LL experiments into one data set. Each MOLLI study consists of three successive LL inversion recovery (IR) experiments with different inversion times (TI) which are performed consecutively within one breath-hold, for a total of 11 images over 17 heartbeats. Images are acquired in late diastole using a single-shot steady-state free-precession (SSFP) technique combined with sensitivity encoding (SENSE) [44]. By combining the three inversions, the relaxation curve is sampled in an interleaved manner, resulting in a sufficient number of points for accurate T1 quantification (Fig. 6) [10]. With some vendors, these data are automatically entered into three-parameter curve fitting at the scanner and T1 times are calculated on a per-pixel basis. To generate the inline T1 map, the acquired IR images are first registered using a motion correction algorithm which is based on estimating synthetic images presenting contrast changes similar to the acquired images solving a variational energy minimisation problem [45] (Fig. 7).

The ShMOLLI sequence requires a short breath-hold [36] and can generate rapid and high-resolution myocardial T1 maps. The imaging time with ShMOLLI is 9.0 ± 1.1 s, compared with the 17.6 ± 2.9 s required with MOLLI. In order to shorten the breath-hold, ShMOLLI does not achieve a full recovery of the longitudinal magnetisation between sequential inversion pulses. ShMOLLI uses a similar effective TI principle to MOLLI but over only nine heartbeats. ShMOLLI has less heart rate dependency, which may improve accuracy. The measurements of myocardial T1 by ShMOLLI are in good agreement with previous measurements using MOLLI [36].

The recently described SASHA sequence [37] uses a single-shot balanced SSFP readout to provide good signal-to-noise ratio and blood–tissue contrast. This approach will overcome the limitations of MOLLI that underestimate T1 values [10, 41, 36] and which are known to have greater underestimation in short T2 tissues such as the myocardium [46]. This sequence consists of ten electrocardiogram-triggered single-shot balanced SSFP images in a breath-hold. The first image is acquired without magnetisation preparation and the remaining nine images follow saturation pulses with variable saturation recovery times [37]. The accuracy of SASHA T1 values is independent of absolute T1, T2, heart rate, flip angle and off-resonant frequencies up to 696 Hs.

In the presence of arrhythmias, a common co-morbidity in patients with heart failure, T1 mapping image quality is usually sub-optimal. Arrhythmia-insensitive inversion recovery sequences have been developed with the purpose of generating a technique insensitive to heart rate variability [38]. The novel preparation pre-pulse, called SAPPHIRE, which consists of a combination of saturation and inversion pulses, is introduced to remove the magnetisation history in each heartbeat and eliminate the need for rest periods in T1 mapping.

### T1 mapping: acquisition protocol and post-processing

**Fig. 8:**
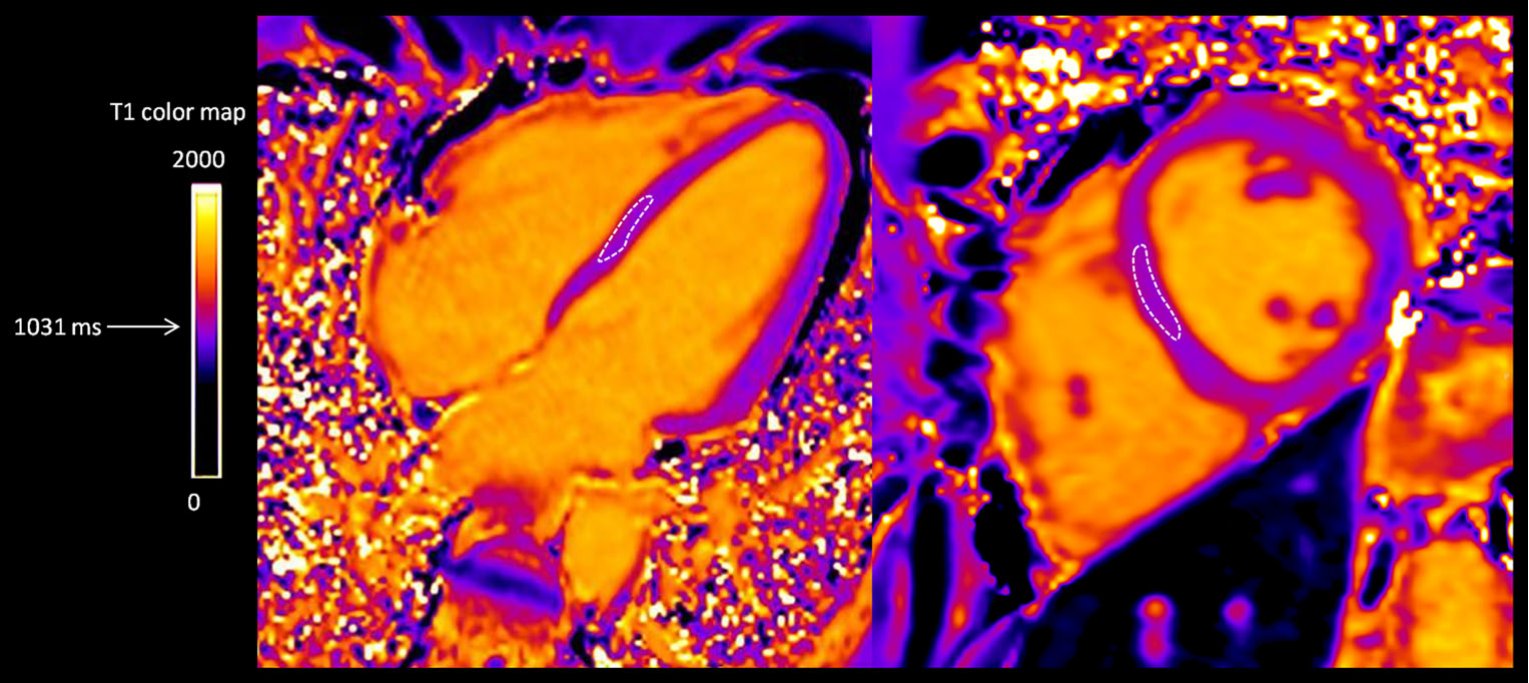
Quantification of native T1 values from a healthy volunteer in a four-chamber plane (**a**) and in a short-axis (**b**) view. A ROI is drawn in the septum. Care is particularly taken to avoid “contamination” with signal from the blood pool and the epicardial fat. Normal values of native T1 at 3 T with MOLLI sequence are 1,031 ± 24 ms. (Reprinted and modified, with permission, from Perea et al. [48])

The most widely used and most extensively validated sequences for T1 mapping are MOLLI-based sequences [10, 42, 47]. Data may be acquired in basal, mid-ventricular and apical short-axis and in four-chamber views. To quantify T1 values, a region of interest (ROI) can be drawn in the septum in a four-chamber plane (excluding areas of focal fibrosis), assuming this to be representative of the whole myocardium. The ROI can be placed, as well, in the short-axis if it is more convenient for avoiding scar areas. The ROI has to be within the myocardium and does not include blood or epicardial fat (Fig. 8) [48] Another strategy to quantify T1 values is segmental analysis, but it is time-consuming and can also be problematic due to ventricular motion artefacts, which occur most frequently in the inferolateral region [49].

### Post-contrast T1 mapping and ECV

**Fig. 9:**
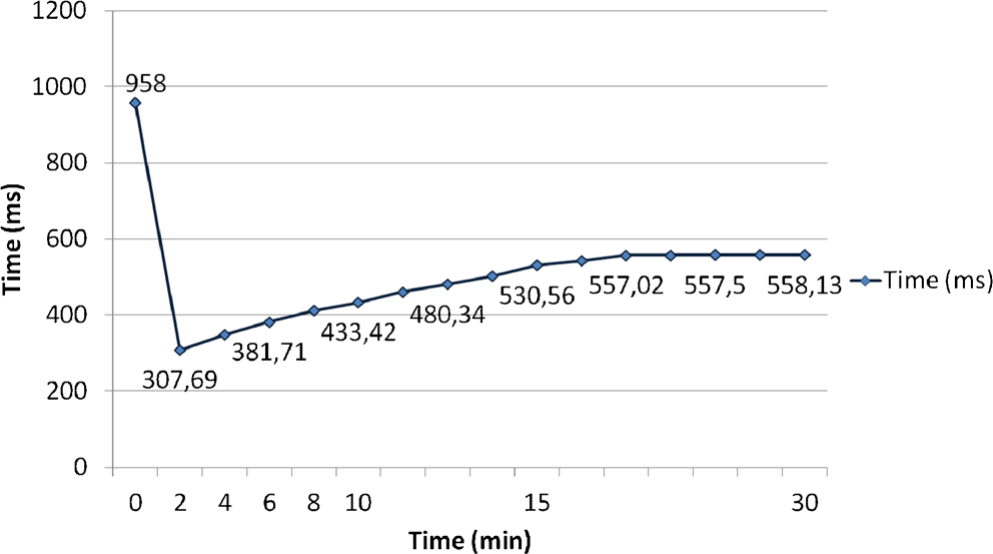
Myocardial T1 values obtained in a healthy volunteer from minute 0 to minute 30 after administration of 0.15 mmol/kg of gadopentetate dimeglumine. T1 values are expressed as means. The exponential recovery of myocardial T1 reflects the washout of the contrast agent from the myocardium

The use of an extrinsic contrast agent adds another dimension to CMR tissue characterisation. The interstitial space can be directly assessed using standard gadolinium chelates. The post-contrast T1 maps are evaluated at different time points after contrast administration and may be used to obtain a curve of myocardial T1 recovery reflecting the contrast agent wash-out [42] (Fig. 9).

At a fixed time after contrast administration, T1 may be reduced in cardiac disease, suggesting increased interstitial space [34]. However, in addition to heart rate and acquisition-related confounders, isolated post-contrast T1 values are influenced by a number of factors, including body fat percentage, reduced renal function, altered haematocrit, native T1, delay time in measurement after contrast administration and gadolinium characteristics (dose, concentration and water exchange rate). Consequently, native T1 mapping and ECV are currently preferred for T1 quantification [35]. If, instead, the ratio of signal change in blood and myocardium after contrast administration is calculated, corrected by the haematocrit, the ECV, which reflects the interstitial space, can be calculated, avoiding confounding factors.

**Fig. 10:**
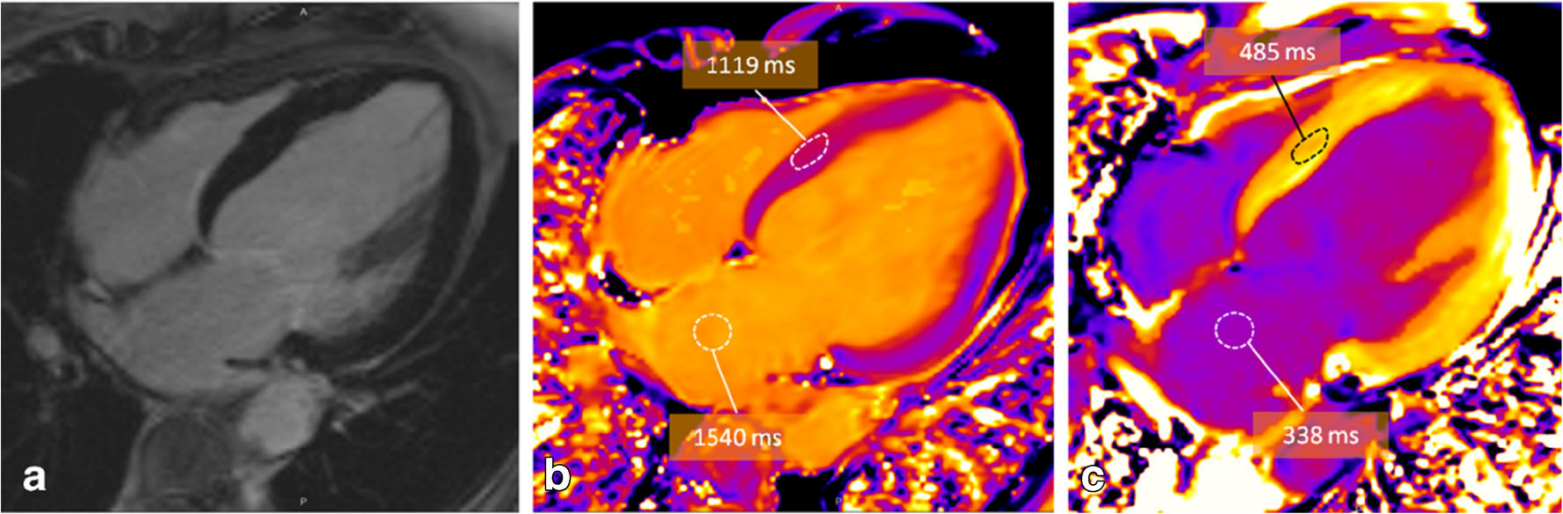
ECV measured using manual ROIs drawn on T1 maps in a patient with idiopathic dilated cardiomyopathy. **a** Four-chamber myocardial delayed enhancement CMR image shows absence of late contrast enhancement. **b**, **c** T1 maps using MOLLI; pre-contrast (**b**) and post-contrast (**c**) showing marked T1 shortening. The two T1 maps are combined with 1-haematocrit blood correction to calculate ECV. Haematocrit in this patient was 38 % and ECV 31 % (slightly elevated, reflecting diffuse fibrosis)

The ECV technique introduces a potentially important new method to examine the myocardium because it is sensitive to the distribution of the left ventricular myocardium into its cellular and ECM compartments. Alterations in these compartments occur from different pathophysiological processes [50]. The ECV reflects the volume fraction of heart tissue that is not taken by cells. ECV may be measured using manual regions of interest (ROIs) drawn on T1 maps (Fig. 10), by performing a manual or semi-automatic image registration of T1 maps [51] or by a fully automated method calculating pixel-wise ECV parametric maps [52], if native and post-contrast T1 images are co-registered, quantified and adjusted for the haematocrit.

Expansion of the ECV represents a non-specific increase in free water in the myocardium and occurs in a variety of pathologies, including focal and diffuse fibrosis, oedema, and amyloidosis. In the absence of amyloid or oedema [53], expansion of the myocardial CVF is responsible for most ECM expansion, which culminates in mechanical, electrical and vasomotor dysfunction.

The myocardial ECV may be estimated from the concentration of extracellular contrast agent in the myocardium relative to the blood in a steady state. The contrast agent distributes between extracellular space and blood plasma such that the relative pre- and post-contrast signal changes measure the myocardial ECV [32, 54]. The measurements are only valid for tissues where contrast agent concentration is in equilibrium (steady state) or dynamic equilibrium (dynamic steady state) with the contrast agent concentration in the blood pool. Following an intravenous injection, contrast agents are continuously cleared from the blood via renal clearance. If the contrast exchange rate between the blood and the tissue of interest is faster than the renal clearance, then the ratio of contrast agent concentration in the tissue and the blood will, after the short initial period, achieve a dynamic equilibrium and remain unchanged over time [32]. By then substituting in the blood contrast volume of distribution (equal to one minus the haematocrit) the myocardial contrast volume of distribution is obtained, reflecting the fraction of the tissue which is interstitial space, also referred to as myocardial ECV. The ECV in the myocardium is then calculated as follows:

$$ {\mathrm{ECV}}_{\mathrm{myo}} = \left[1-\mathrm{haematocrit}\right] \times \varDelta \mathrm{R}{1}_{\mathrm{myo}}/\varDelta \mathrm{R}{1}_{\mathrm{blood}} $$

Where ΔR1 is (1/T1 pre-contrast – 1/T1 post-contrast)

Equilibrium distribution can be achieved with a primed contrast infusion (equilibrium contrast-CMR [EQ-CMR]) [55] or might be approximated by the dynamic equilibration achieved by delayed post-bolus measurement [56, 57].

EQ-CMR is a robust, non-invasive method to quantify diffuse myocardial fibrosis, which has been validated against the current gold standard of surgical myocardial biopsy CVF quantification in patients with aortic stenosis and hypertrophic cardiomyopathy [55]. EQ-CMR is achieved by primed infusion (a loading bolus of 0.1 mmol/kg followed by a slow continuous infusion of 0.001 mmol/kg/min [equivalent to 0.1 mmol/kg over 90 min]) [55]. Standard LGE imaging is possible after the bolus, although the sensitivity of LGE may decline since doses as low as 0.1 mmol/kg have reduced sensitivity for myocardial infarction. This technique is time-consuming, but continuous infusion removes contrast kinetic effects, measuring diffuse fibrosis in vivo.

The bolus only technique assumes that at a sufficient time after a single-contrast bolus, a dynamic equilibrium exists [56] allowing the equivalent ECV measurement. Post-gadolinium ECV is stable from approximately 8.5 min after administration of a bolus and remains at a steady state up to 50 min after gadolinium injection [56, 58] The contrast dose varies across groups (0.15 [51] or 0.2 [56] mmol/kg), enabling a quality LGE imaging 10–15 min after bolus. ECV can be measured with simple gadolinium contrast bolus as accurately as with an infusion, but with slightly less precision [56]. The bolus strategy to measure myocardial ECV is preferred against the primed infusion because it simplifies the data acquisition protocol and facilitates its integration into routine CMR practice. This technique has been validated histologically in distinct disease groups and the correlation with CVF is similar to that with the infusion technique and does not differ statistically [57]. Bolus only is sufficient for ECV measurement across a range of cardiac diseases. However, when ECV is >40 % (amyloid, LGE areas of hypertrophic cardiomyopathy and myocardial infarction), the bolus only technique consistently measures ECV higher compared with infusion, therefore, in selected cases (especially amyloidosis) the infusion method is preferred.

T1 values are affected by confounding variables mentioned before. Due to these factors, T1 times cannot be readily compared to T1 data from other centres. There are different normal T1 values in the literature depending on the field strength, the scanner manufacturer, the kind of sequence and other parameters related with the acquisition protocol and the post-processing [36, 58, 59, 49]. In order to use native myocardial T1 mapping to accurately identify disease states, it is advisable to obtain the reference values in each scenario by performing a study with healthy volunteers. In contrast, ECV is an inherent physiological property that should not be affected by these variables. The ECV data of normal volunteers do not significantly differ between the different studies, being in the range of 24–28 % [56, 58, 59, 60, 61, 62]; *see* Table 2.

**Table 2:** Normal values of myocardial ECV at different studies

	Field strength	Scanner	Contrast	Technique	Dose (mmol/kg)	T1 mapping sequence	ECV (%)
Broberg et al. [60] 2010	1.5 T/*3 T*	Achieva/Intera, Philips	Gadodiamida	Only bolus	0.15	Look-Locker	24.8
Schelbert et al. [56] 2011	1.5 T	Espree, Siemens	Gadoteridol	Only bolus	0.2	MOLLI	24.1
Lee et al. [58] 2011	3 T	Verio, Siemens	Gadopentetate Dimeglumine	Only bolus	0.15	MOLLI	26.7
Kellman et al. [61] 2012	1.5 T	Avanto/Espree, Siemens	Gd-DTPA	Only bolus	0.15	MOLLI	25.4
Sado et al. [85] 2012	1.5 T	Avanto, Siemens	Gadoterate Meglumine	Bolus + infusion	0.1 + 0.002/min^a^	FLASH IR	25.3
Salerno et al. [62] 2013	1.5 T	Avanto, Siemens	Gd-DTPA	Bolus + infusion	0.1 + 0.001/min^b^	MOLLI	28.5
Liu et al. [86] 2013	1.5 T	Avanto/Espree, Siemens	Gadopentetate Dimeglumine	Only bolus	0.15	MOLLI	26.9

^a^Bolus of 0.1 mmol/kg followed by infusion of 0.002 mmol/kg/min

^b^Bolus of 0.1 mmol/kg followed by infusion of 0.001 mmol/kg/min

## Clinical applications of interstitial imaging

**Fig. 11:**
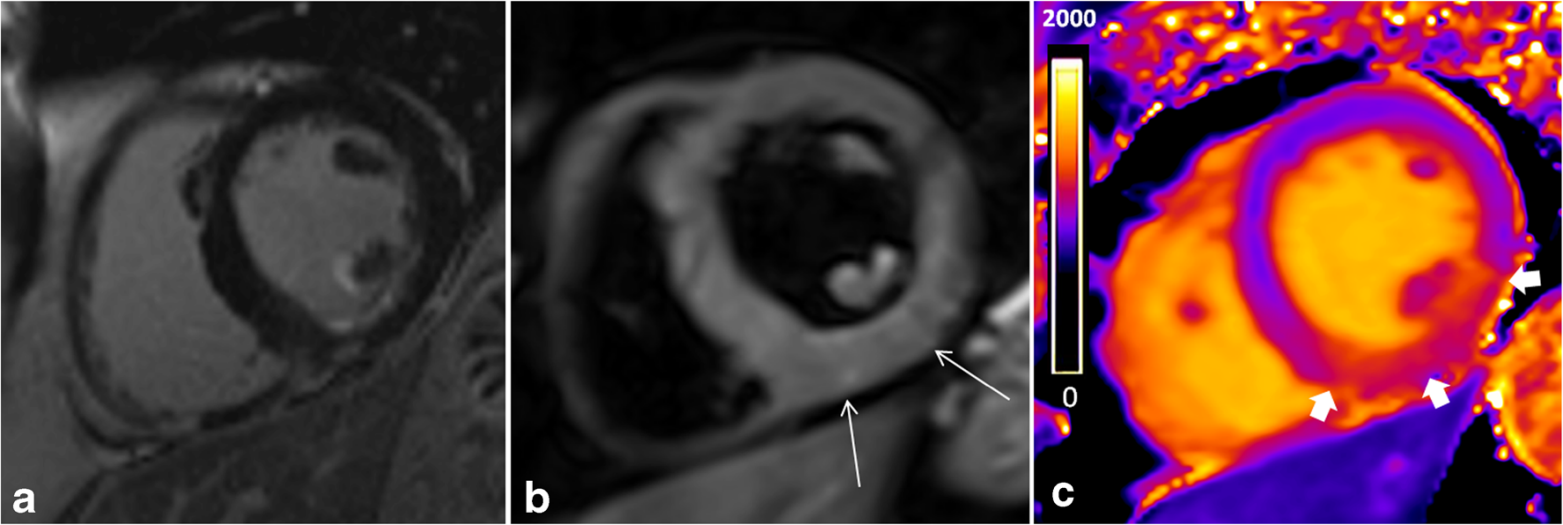
T1 mapping in acute myocardial infarction. Subendocardial enhancement (**a**) in the inferolateral, midventricular segment of the left ventricle. Although the T2-weighted images (**b**) show only a mild increase in brightness (*long arrows*), there is an area of increased T1 values (1,208 ms, into the *orange* range of the colour scale) (*short arrows*) (**c**) exceeding the area of LGE enhancement

**Fig. 12:**
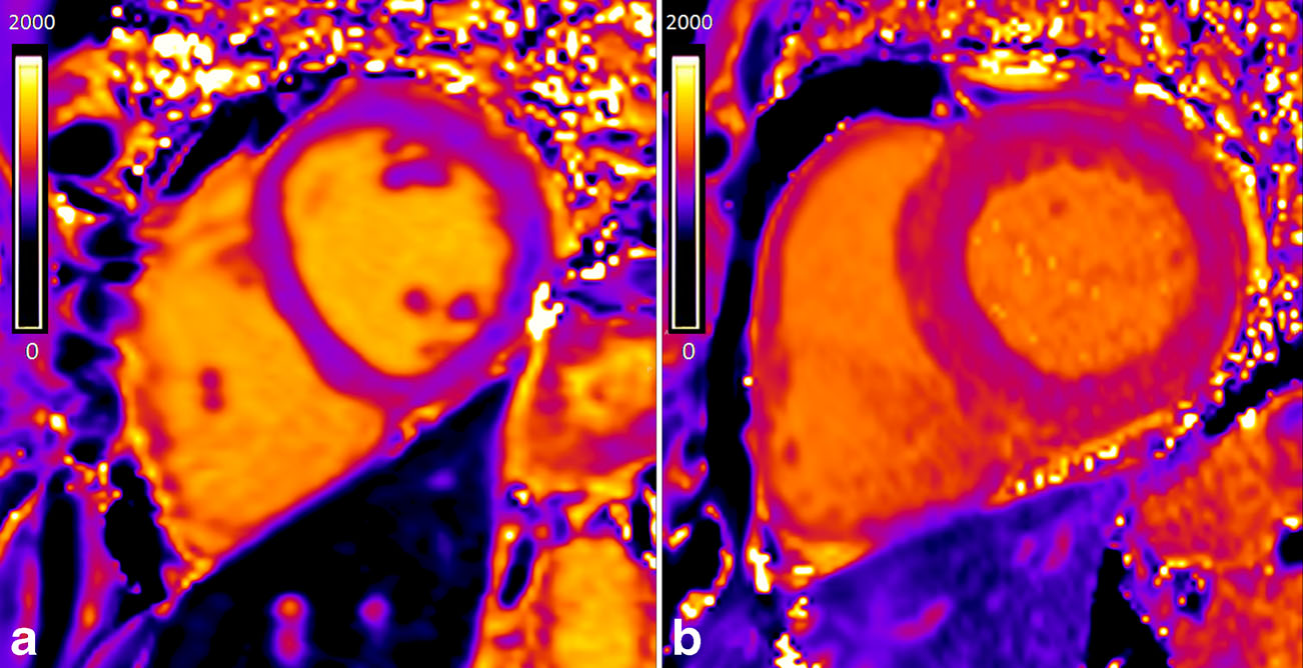
T1 mapping in amyloidosis. MOLLI non-contrast T1 map in a normal volunteer (**a**), and cardiac amyloid patient (**b**). Note the markedly elevated myocardial T1 time in the cardiac amyloid patient (1,195 ms, into the *orange* range of the colour scale) compared with the normal control (1,048 ms, in *purple* range of the scale)

Native T1 distinguishes normal from abnormal myocardium, indicating myocardial disease involving both the myocyte and interstitium. Measurement requires no exogenous contrast administration, making it feasible in patients with severe renal dysfunction or pregnancy. Cardiac T1-mapping without the use of a GBCA has been shown to be sensitive to a variety of pathologies where increased water is present, such as oedema [63, 64], focal or diffuse fibrosis [65] and amyloidosis [66]. Acute myocardial injury is accompanied by intracellular and interstitial oedema and is traditionally detected by increased T2 signal, although pre-contrast T1 mapping may prove to be equally effective and robust [64]. The oedema in myocardial ischaemia and infarction can be recognised by increases in T1 with high sensitivity and specificity [67, 68, 69] (Fig. 11). In chronic myocardial infarction, there is replacement of myocardial cells by fibrosis with an increase in extracellular collagen. Consequently, T1 values are higher than in normal myocardium, but not as high as in acute myocardial infarction [67]. For determining the area at risk, native T1 and T2 mapping provide similar results and closely match the area at risk as defined by microspheres in animal models [64]. Native T1 mapping is superior compared with T2-weighted and LGE techniques in detecting acute myocarditis [70, 71], which is helpful in subtle focal disease [72] and may detect pathology missed by LGE technique, such as pan-myocarditis [72]. Native T1 values provide diagnostic accuracy to discriminate between normal and diffuse fibrosis in patients with non-ischaemic dilated cardiomyopathies [73, 74] and hypertrophic cardiomyopathy [73, 74], having the potential to become a test in patients with suspected diffuse fibrosis, which may be missed by classic LGE imaging. Furthermore, native T1 is significantly elevated in patients with aortic stenosis and correlates with the CVF quantified at biopsy [65]. Diffuse fibrosis is an important clinical parameter in aortic stenosis and is also reflected in the degree of postoperative recovery. However, fibrosis is a potentially reversible phenomenon under several therapies [75]. Cardiac amyloidosis shows markedly increased non-contrast T1 relaxation times in the myocardium [66, 76], even more pronounced that in aortic stenosis [66] (Fig. 12). Myocardial T1 mapping is an accurate technique for the detection of cardiac involvement in amyloidosis, avoiding the administration of GBCA that frequently is problematic in this group of patients [66]. Other pathologies may result in a decrease of native T1 values, like Anderson-Fabry disease, because of the intracellular lipid accumulation [77], and iron overload where T1 mapping is superior to the classic T2* sequence for the detection of early iron overload [78].

**Fig. 13:**
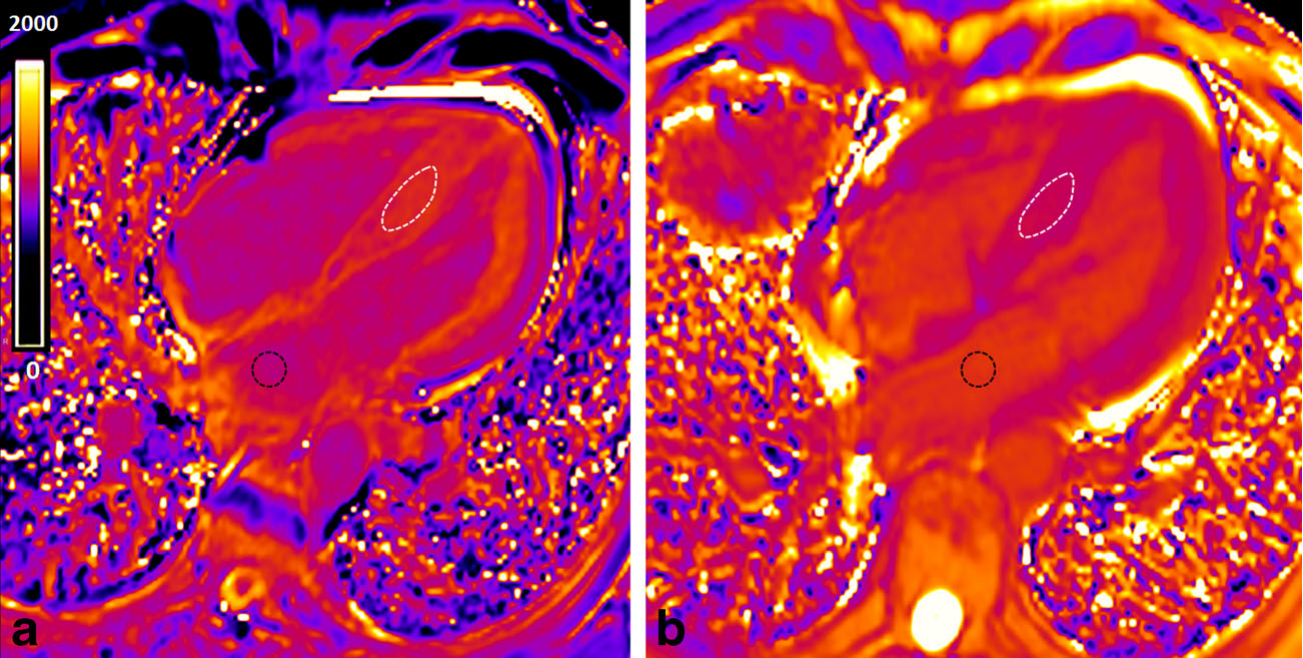
Native (**a**) and post-contrast with the EQ-CMR technique (**b**) T1 maps in a patient with AL amyloidosis. A ROI is placed in the septum (*white ROI*) and in the blood (*black ROI*) in the native and post-contrast T1 maps. Native T1 value is increased (1,219 ms; *N*, 1,031 ± 24) and post-contrast T1 time is shortened (481 ms). The two T1 maps values combined with 1-haematocrit blood correction enable the calculation of the ECV that is increased (68 %) in this patient. (Native blood T1, 1,121 ms; post-contrast blood T1, 541 ms; haematocrit, 48 %)

The use of GBCA allows a direct measurement of the size of the extracellular space, reflecting interstitial disease. The values of myocardial ECV are increased in cardiac diseases (hypertrophic [55] and dilated [79] cardiomyopathy, aortic stenosis [55], infarction [51], diabetes [80] and congenital heart diseases with myocardial dysfunction [60]) reflecting diffuse myocardial fibrosis. In myocarditis, where expansion of ECV is due to oedema/inflammation/necrosis, the ECV quantification with LGE imaging improve the diagnostic accuracy of CMR compared with standard “Lake Louise” criteria [81]. Expansion of the myocardial ECV in amyloidosis is higher than in any other disease generating diagnostic specificity above a certain threshold [53] (Fig. 13). Myocardial ECV measurement has the potential to become the first non-invasive test to quantify cardiac amyloid burden.

These new techniques might help us to detect subclinical myocardial changes in cardiovascular risk populations that otherwise could be missed by traditional imaging techniques, enabling an improvement in therapeutic strategies, monitoring the treatment effect and improving clinical outcome.

## Conclusions

The T1 mapping techniques and ECV imaging by CMR appear to be sufficiently robust methods for diagnosis of many cardiac diseases. Just as native T1 mapping may be considered an intrinsic myocardial contrast, the ECV after GBCA is a direct measurement of the size of the extracellular space, reflecting interstitial disease. This technique separates the myocardium into its cellular and interstitial components. These techniques promise early detection of the disease and have the potential to provide a more individualised therapy. Consequently, native T1 mapping and ECV might supply a CMR biomarker for myocardial fibrosis, justifying their use in clinical practice. However, more research is required before a large-scale application for clinical decision-making can be recommended.

## Abbreviations

CMR: cardiac magnetic resonance
LGE: late gadolinium enhancement
ECM: extracellular matrix
CVF: collagen volume fraction
GBCA: gadolinium based contrast agents
ECV: extracellular volume fraction
MOLLI: modified Look-Locker inversion recovery
ShMOLLI: shortened modified Look-Locker inversion recovery
SASHA: saturation recovery single-shot acquisition
SAPPHIRE: saturation pulse prepared heart rate independent inversion recovery
LL: Look-Locker
IR: inversion recovery
SSFP: steady-state free-precession
SENSE: sensitivity encoding
TI: inversion times
ROI: region of interest
EQ-CMR: equilibrium contrast-CMR
